# Supplementary material for: Effects of green seaweed extract on Arabidopsis early development suggest roles for hormone signalling in plant responses to algal fertilisers
Source: Sci Rep. 2019 Feb 13;9:1983. doi: 10.1038/s41598-018-38093-2 (PMC6374390; doi:10.1038/s41598-018-38093-2)
Supplement: Supplementary file 1 — Supplemental Figures [file 41598_2018_38093_MOESM1_ESM.pdf]

Supplemental data for:

Effects of green seaweed extract on *Arabidopsis* early development suggest roles for hormone signalling in plant responses to algal fertilisers.

Fatemeh Ghaderiardakani, Ellen Collas, Deborah Kohn Damiano, Katherine Tagg, Neil S. Graham and Juliet C. Coates.

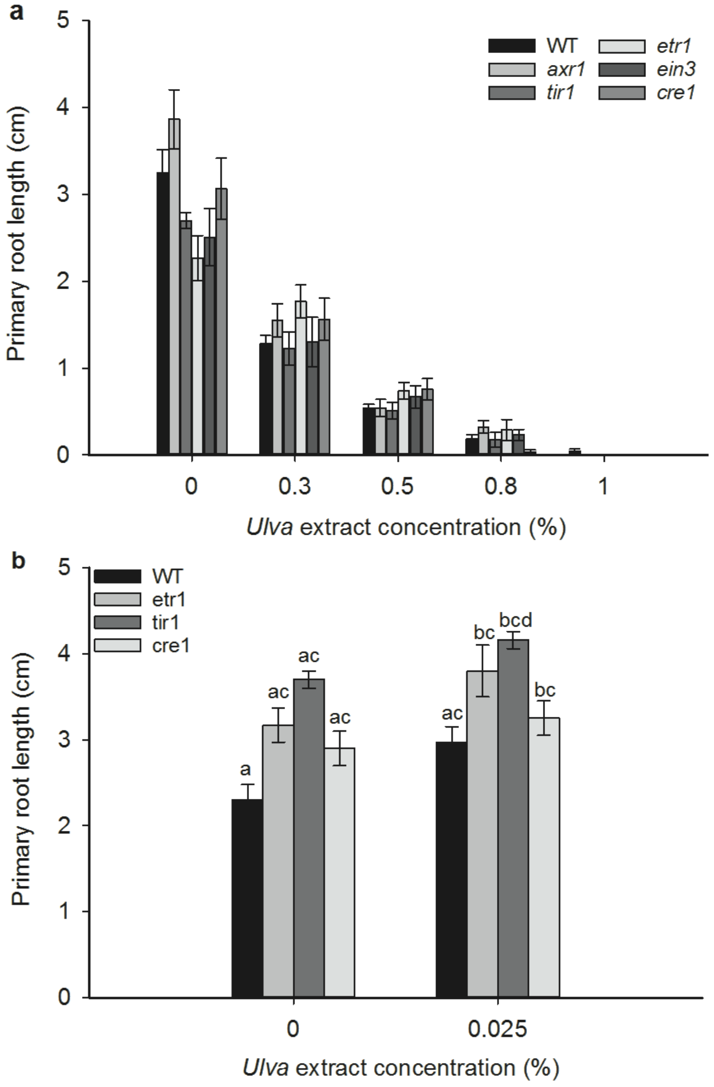

Supplemental Figure 1. Ethylene, auxin and cytokinin signaling mutants' root growth is inhibited by high concentrations of *Ulva* extract, similarly to wild-type plants.
a) Inhibition of root growth by 0.3-1% *Ulva* extract in all hormone mutants, similarly to wild type. Comparison across genotypes and treatments using a Kruskal-Wallis test and a Dunn's post-hoc test indicates that 0.3% *Ulva* extract reduces root growth ($p \leq 0.05\%$) compared to the control in all genotypes but *etr1*, while 0.5% reduces root growth ($p \leq 0.05\%$) compared to the control in all genotypes but *cre1*. 0.8% and 1% *Ulva* extract significantly reduce root growth in all genotypes compared to the control ($p \leq 0.05$). In terms of between-genotype differences, under control conditions only *etr1* is significantly different from other genotypes ($p<0.05$), at 0.3% and 0.5% there are no significant differences between the genotypes, at 0.8% *cre1* is significantly different from wild-type and *axr1* ($p<0.05$) and at 1% no genotypes are significantly different from one another.
b) Stimulation of root growth in all hormone signaling mutants by 0.025% *Ulva* extract. An analysis of variance followed by a Tukey's post-hoc test demonstrates significant differences between wild-type control and *etr1* and *tir1* treated with *Ulva* extract ($p<0.01$), between wild type control and cre1 treated with *Ulva* extract ($p<0.05$), between wild-type treated with *Ulva* extract and and *tir1* treated with *Ulva* extract ($p<0.05$), between *cre1* control plants and *etr1* treated with *Ulva* extract, and between *cre1* control and *tir1* treated with *Ulva* extract. These differences are represented by the letters above the graph.

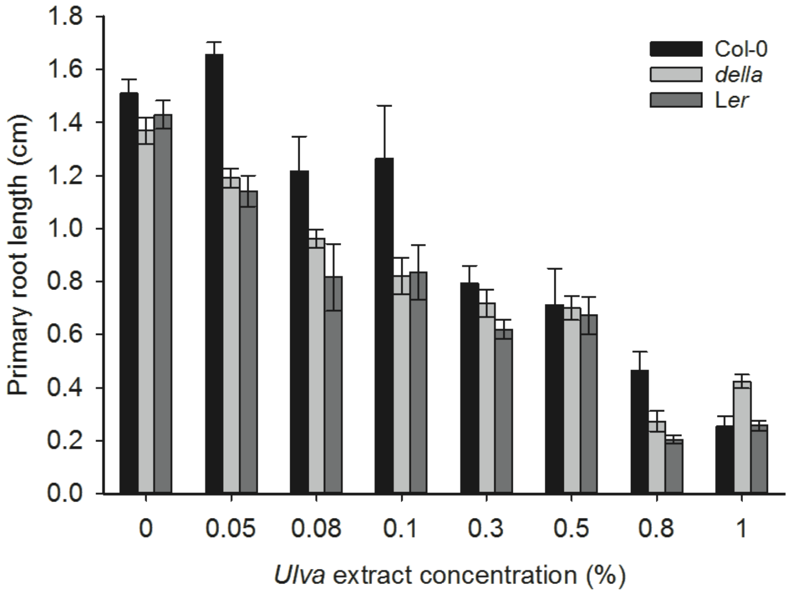

Supplemental Figure 2. The *Arabidopsis* quintuple *della* mutant is sensitive to the inhibitory effects of *Ulva* extract on root growth.

The *della* mutant was generated in a L*er* background also containing 3Mb of Col-0 sequence (Belfield et al., 2012). For each *Ulva* extract concentration, the effects on Col-0, *della* and L*er* are shown.

Supplemental Reference

Belfield, E. J. *et al.* Genome-wide analysis of mutations in mutant lineages selected following fast-neutron irradiation mutagenesis of Arabidopsis thaliana. *Genome research* **22**, 1306-1315 (2012).
